# Comparative performance of mFI‐5 and mFI‐11 thresholds in predicting outcomes after holmium laser enucleation of the prostate

**DOI:** 10.1002/bco2.70244

**Published:** 2026-07-08

**Authors:** Hasim Bakbak, Gurpremjit Singh, Ahmad Abdelaziz, Adam Williams, Juanita Velasquez Ospina, Jonathan E. Katz, Robert Marcovich, Alan Jerome Wein, Hemendra N. Shah

**Affiliations:** ^1^ Desai Sethi Urology Institute University of Miami Miller School of Medicine Miami Florida USA

**Keywords:** benign prostatic hyperplasia, complications, elderly, enlarged prostate, frailty, HoLEP, octogenarians

## Abstract

**Objectives:**

This work aimed to evaluate the safety and efficacy of holmium laser enucleation of the prostate across different degrees of frailty using the 11‐item and 5‐item modified frailty indices and to compare perioperative and postoperative outcomes across multiple frailty thresholds.

**Materials and Methods:**

We retrospectively reviewed patients who underwent HoLEP at our institution between July 2017 and August 2025. Frailty was assessed using mFI‐11 (cutoffs ≥0.18, ≥0.27, ≥0.36) and mFI‐5 (≥0.40). Perioperative variables, functional outcomes, and postoperative complications were compared. Agreement between frailty definitions was assessed using Cohen's kappa. Logistic regression was used to evaluate complication risk.

**Results:**

Among 857 patients, overall frailty burden was low ranging from 16.2% with mFI‐11 ≥0.36 to 52.6% with mFI‐11 ≥0.18, and 27.5% by mFI‐5 ≥0.40. Functional outcomes improved in all groups, though patients defined as frail by mFI‐11 ≥0.36 demonstrated less improvement in IPSS at 1 year and lower Qmax at select time points. Major complications were uncommon and did not demonstrate a consistent frailty gradient. Transfusion risk was higher only at mFI‐11 ≥0.36. Urinary tract infection showed a dose–response relationship with increasing mFI‐11, with approximately a 20% increase in odds per one‐point increase.

**Conclusions:**

HoLEP is safe and effective across frailty definitions. Frailty cutoff selection meaningfully influences risk stratification, with mFI‐11 ≥0.36 providing the most clinically informative discrimination.

## 1 INTRODUCTION

Benign prostatic enlargement is the most common benign neoplasm in men and remains the leading cause of lower urinary tract symptoms (LUTS), affecting nearly three‐quarters of men in their seventh decade of life.^1^ The introduction of medical therapy for benign prostatic obstruction (BPO) in the 1990s represented a paradigm shift, providing effective nonsurgical alternatives for patients who had previously relied almost exclusively on operative intervention.^2^ However, increasing life expectancy and prolonged medical management have resulted in patients presenting for procedural intervention at older ages and with larger prostate volumes.^3^ Globally, rising longevity has expanded the elderly male population, accompanied by parallel increases in comorbidity burden and benign prostatic hyperplasia (BPH) prevalence.^4^ As populations age, the intersecting effects of BPH, multimorbidity, and functional decline have become central considerations in treatment selection.^5^, ^6^ Importantly, chronological age alone is an imperfect surrogate for physiologic resilience.^7^ Frailty, reflecting diminished physiologic reserve, offers a more accurate framework for risk stratification.^7^ Notably, age and frailty do not overlap completely, with a substantial proportion of individuals in their eighth decade remaining nonfrail.^8^

Frailty carries meaningful implications for both disease progression and therapy tolerance. It has been linked to more rapid worsening of BPH symptoms and increased vulnerability to medication‐related adverse effects such as orthostatic hypotension, falls, and cognitive changes.^9^, ^10^ Medication adherence is also poorer among frail individuals, further reducing therapeutic benefit.^11^ Earlier studies identified frailty as a predictor of inferior surgical outcomes, and subsequent work has shown delayed postoperative recovery in this population.^12^, ^13^ Despite its clinical value, frailty classification remains inconsistent across studies using the 11‐item modified frailty index (mFI‐11), with thresholds ranging from ≥2 deficits (≥0.18) to ≥3 (0.27) or ≥4 (≥0.36), contributing to heterogeneity in the literature.^14^, ^15^, ^16^

Transurethral resection of the prostate (TURP) has long been regarded as the gold‐standard surgical therapy for BPH and remains one of the most frequently performed procedures for bladder outlet obstruction in frail men in the United States.^17^ Although frail patients experience meaningful improvement in voiding symptoms by 6 months after TURP, their symptom relief is consistently less pronounced than that observed in nonfrail individuals.^18^ Over the past two decades, holmium laser enucleation of the prostate (HoLEP) has gained prominence as a size‐independent surgical option and is recommended as a preferred option in medically complicated patients by contemporary AUA guidelines.^19^, ^20^ HoLEP has demonstrated durable safety and efficacy across a range of high‐risk groups, including elderly patients and those with elevated ASA scores or medical vulnerability.^21^, ^22^ Notably, in frail individuals, HoLEP is associated with fewer complications compared to TURP.^17^

Despite these advantages, data specifically examining HoLEP outcomes in frail patients remain sparse, and most prior studies have relied on the mFI‐5 or other abbreviated frailty tools rather than the more comprehensive mFI‐11.^23^, ^24^ To date, no study has evaluated HoLEP outcomes stratified by mFI‐11, compared mFI‐11 with mFI‐5, or examined different frailty cutoff thresholds within the same HoLEP cohort. Accordingly, the present study evaluates the safety and efficacy of HoLEP across varying degrees of frailty using both mFI‐5 and multiple mFI‐11 thresholds.

## 2 METHODS

### 2.1 Study design

We retrospectively identified patients who underwent HoLEP at our tertiary academic centre between July 2017 and August 2025 using a prospectively maintained institutional HoLEP registry. Frailty was quantified using the modified frailty index (mFI), derived from the Canadian Study of Health and Aging Frailty Index, and validated across various surgical populations.^14^ Both mFI‐11 and mFI‐5 were used. For mFI‐11, the following 11 variables were captured: functional dependence, diabetes mellitus (DM), chronic obstructive pulmonary disease (COPD), congestive heart failure (CHF), hypertension (HT), prior myocardial infarction (MI), prior percutaneous coronary intervention (PCI), prior cardiac surgery or angina, peripheral vascular disease (PVD), impaired sensorium, prior transient ischemic attack (TIA) or cerebrovascular accident (CVA), and anticoagulation use or bleeding disorder. For mFI‐5, the five components were functional dependence, DM, COPD, CHF, and HT. Diabetes was captured in accordance with mFI definitions and was not subclassified by type; given the age profile of the cohort, most cases were expected to represent type 2 diabetes. Frailty cutoffs were applied as mFI‐11 ≥0.18, ≥0.27, and ≥0.36, and mFI‐5 ≥0.40, consistent with literature. Patients were classified as frail or nonfrail using these multiple frailty indices and prespecified cutoffs. Baseline demographics, perioperative outcomes, functional outcomes, and complications were compared between frail and nonfrail groups. We report this study in accordance with the STROBE guidelines. Institutional Review Board approval was obtained before data collection and analysis (IRB #20180511).

### 2.2 Preoperative workup

Prostate size was determined using imaging obtained within 12 months prior to surgery. Patients with elevated PSA underwent additional evaluation with 4Kscore testing, mpMRI, and/or prostate biopsy based on shared decision‐making. Anticoagulant and antiplatelet agents were held preoperatively when feasible in coordination with the patient's primary care physician or cardiologist. Urine culture was obtained within 2 weeks of the scheduled procedure, and patients with positive cultures received culture‐directed antibiotics before surgery.

### 2.3 Procedure

All cases were performed by a single surgeon under general anaesthesia in the lithotomy position using an en‐bloc HoLEP technique as previously described.^25^ In recent years, our surgical template was modified, when possible, to preserve the anterior fibromuscular stroma and overlying mucosa. Enucleation was performed using a 26‐French laser resectoscope and a 550‐μm laser fibre connected to a 100‐W or 120‐W holmium: YAG laser platform, incorporating Moses pulse modulation technology when available. Enucleated tissue was morcellated intravesically using a mechanical morcellator (VersaCut™ or Piranha™). At the conclusion of the procedure, a three‐way 22‐French Foley catheter was placed without traction and continuous bladder irrigation was initiated.

### 2.4 Postoperative management

All patients were admitted overnight for observation and continuous bladder irrigation. On postoperative day 1 (POD 1), complete blood count and basic metabolic panel were obtained, followed by a voiding trial. Patients with satisfactory voiding were discharged. Patients who failed the trial or had a post‐void residual (PVR) > 150 mL were discharged with an indwelling catheter and scheduled for a repeat outpatient trial within 2–7 days. Patients with chronic urinary retention and PVR > 500 mL who were not catheter‐dependent preoperatively were typically discharged with an indwelling Foley catheter for 1–3 weeks. All prostate‐directed medications were discontinued immediately after surgery.

### 2.5 Postoperative follow‐up and outcomes

Follow‐up evaluations were performed at 6 weeks and at 3, 6, and 12 months. Functional outcomes were assessed using the International Prostate Symptom Score (IPSS), maximum urinary flow rate (Qmax), and post‐void residual urine volume (PVR). PSA nadir was recorded at approximately 3 months when available. Postoperative complications were systematically captured, including transfusion, acute urinary retention (AUR), urinary tract infection (UTI) requiring antibiotics, urethral stricture, bladder neck contracture (BNC), and transient urinary incontinence (TUI). Urinary incontinence was defined as any involuntary leakage requiring pad use at any postoperative time point, including stress and urge incontinence.

### 2.6 Data collection and study variables

Data extracted included baseline demographics, comorbidities, anticoagulation or antiplatelet use, prostate size, PSA, serum creatinine, and preoperative urinary parameters including the International Prostate Symptom Score (IPSS), maximum urinary flow rate (Qmax), and post‐void residual urine volume (PVR). Frailty was assessed using both mFI‐11 and mFI‐5 and frailty cutoffs were applied as mFI‐11 ≥0.18, ≥0.27, and ≥0.36, and mFI‐5 ≥0.40. Operative and perioperative variables included operative duration, enucleated tissue weight, perioperative laboratory results including haemoglobin change, POD 1 haemoglobin and creatinine, transfusion, catheterization, and length of stay. Operation time was defined as the total time spent by the patient in the operation room.

### 2.7 Statistical analysis

Continuous variables were summarized as medians with interquartile ranges, and categorical variables as counts with percentages. Within each frailty definition and cutoff, comparisons between frail and nonfrail groups were performed using the Wilcoxon rank‐sum test for continuous variables and Fisher exact or chi‐square tests for categorical variables, as appropriate. Agreement was assessed using Cohen's kappa. For postoperative complications, we used odds ratios, and trend across increasing mFI‐11 cutoffs was evaluated using the Cochran–Armitage test. Logistic regression was used to model complication outcomes using continuous frailty scores. Diagnostic performance of alternative frailty thresholds was evaluated using sensitivity, specificity, and Youden's *J* statistic. Statistical significance was defined as a two‐sided *p* value ≤ 0.05. Analyses were performed in R (R Foundation for Statistical Computing, Vienna, Austria).

## 3 RESULTS

A total of 857 patients underwent HoLEP during the study period. Overall frailty burden was low, with 608 patients (70.9%) having an mFI‐11 score ≤2 and 621 (72.5%) having an mFI‐5 score ≤1. HT was the most prevalent frailty component, present in 559 of 857 patients (65.2%), followed by anticoagulation in 343 of 857 (40.0%) and DM in 216 of 857 (25.2%). Together, HT, DM, and anticoagulation comprised 1118 of 1645 total deficits (68.0%), accounting for more than two‐thirds of the cumulative frailty burden in the cohort (Table 1).

**TABLE 1:** Distribution of frailty scores and components, *n* (% of total deficits).

Prevalence of individual mFI component number of patients (%)	
Hypertension	559 (34.0%)
Anticoagulation or bleeding disorder	343 (20.9%)
Diabetes mellitus	216 (13.1%)
Myocardial infarction	177 (10.8%)
Percutaneous coronary intervention, cardiac surgery, or angina	77 (4.7%)
Transient ischemic attack or cerebrovascular accident	69 (4.2%)
Peripheral vascular disease	59 (3.6%)
Functional dependence	50 (3.0%)
Chronic obstructive pulmonary disease	49 (3.0%)
Chronic heart failure	31 (1.9%)
Impaired sensorium	15 (0.9%)
Total	1645

Distribution of frailty score		
Total mFI score	mFI‐11: *N* (%)	mFI‐5: *N* (%)
0	175 (20.4%)	250 (29.2%)
1	231 (27.0%)	371 (43.3%)
2	202 (23.6%)	181 (21.1%)
3	110 (12.8%)	48 (5.6%)
4	66 (7.7%)	7 (0.8%)
5	39 (4.5%)	0 (0%)
6	22 (2.6%)	—
7	8 (0.9%)	—
8	3 (0.4%)	—
9	1 (0.1%)	—
10	—	—
11	—	—

Using the mFI‐11, the proportion classified as frail ranged from 16.2% at a cutoff of ≥0.36 to 29.1% at ≥0.27 and 52.6% at ≥0.18. In contrast, application of the mFI‐5 identified 27.5% of patients as frail at a cutoff of ≥0.40. Across all frailty definitions, frail patients were significantly older. In contrast, nonfrail individuals consistently had higher baseline PSA than frail individuals. Preoperative haemoglobin was significantly lower in extremely frail patients with frailty defined by mFI‐11 ≥0.36. Catheter dependency was significantly more common in all frail patients but failed to reach statistical significance when frailty was defined by mFI‐11 ≥0.36 (Table 2a). Agreement varied across frailty definitions, with substantial agreement between mFI‐11 ≥0.36 and ≥0.27 (*κ* 0.6–0.8), moderate agreement for most comparisons (*κ* 0.4–0.6), and fair agreement between mFI‐11 ≥0.36 and ≥0.18 (*κ* 0.2–0.4); no comparison achieved near‐perfect agreement (*κ* > 0.80) (Table 2b).

**TABLE 2a:** Baseline characteristics across different frailty cutoffs and indices.

	mF11 ≥ 0.36			mF11 ≥ 0.27			mF11 ≥ 0.18			mF5 ≥ 0.4		
	Frail 139 (16.2%)	Nonfrail 718 (83.8%)	*p*	Frail 249 (29.1%)	Nonfrail 608 (70.9%)	*p*	Frail 451 (52.6%)	Nonfrail 406 (47.4%)	*p*	Frail 236 (27.5%)	Nonfrail 621 (72.5%)	*p*
Age (years)	73 [67–79]	69 [62–75]	**0.01**	72 [65–78]	68 [62–74]	**0.01**	71 [64–77]	68 [61–73]	**0.01**	71 [65–77]	69 [62–75]	**0.01**
BMI (kg/m^2^)	27 [24–30]	27 [25–31]	0.19	27 [25–30]	27 [25–31]	0.54	27 [25–31]	27 [25–30]	0.28	27 [25–31]	27 [25–30]	0.23
PSA (ng/mL)	3.7 [1.5–6.8]	4.7 [2.5–8.4]	**0.01**	4.0 [1.8–7.0]	4.8 [2.5–8.6]	**0.01**	4.2 [2.1–7.7]	5.0 [2.6–8.6]	**0.03**	4.3 [1.7–7.5]	4.7 [2.5–8.4]	**0.05**
Prostate volume (cc)	100 [72–157]	105 [74–154]	0.57	100 [73–152]	105 [75–156]	0.36	103 [74–156]	101 [75–154]	0.87	105 [73–170]	101 [75–150]	0.39
Haemoglobin (g/dL)	13.8 [12.8–15.0]	14.2 [13.2–15.1]	**0.03**	14.0 [12.8–15.2]	14.2 [13.2–15.1]	0.36	14.0 [12.9–15.1]	14.3 [13.3–15.1]	0.06	14.0 [12.8–15.1]	14.2 [13.2–15.1]	0.28
Creatinine (mg/dL)	1.05 [0.90–1.23]	1.04 [0.90–1.20]	0.54	1.03 [0.90–1.23]	1.04 [0.90–1.19]	0.83	1.04 [0.90–1.23]	1.04 [0.90–1.18]	0.36	1.03 [0.90–1.23]	1.05 [0.91–1.20]	0.77
PVR ≥ 300 mL	32 (23.0%)	116 (16.2%)	0.07	52 (20.9%)	96 (15.8%)	0.09	82 (18.2%)	66 (16.3%)	0.47	40 (16.9%)	108 (17.4%)	0.92
Catheter dependency	51 (36.7%)	219 (30.5%)	0.16	95 (38.1%)	175 (28.8%)	**0.01**	163 (36.1%)	107 (26.4%)	**0.01**	97 (41.1%)	173 (27.9%)	**0.01**
Performing CIC	16 (11.5%)	59 (8.2%)	0.25	27 (10.8%)	48 (7.9%)	0.18	40 (8.9%)	35 (8.6%)	0.90	24 (10.2%)	51 (8.2%)	0.42
Active surveillance (GG1)	6 (4.3%)	28 (3.9%)	0.81	10 (4.0%)	24 (3.9%)	1	21 (4.6%)	13 (3.2%)	0.30	12 (5.0%)	22 (3.5%)	0.33

*Note*: Bold values are the *p* values that were statistically significant (defined as *p* ≤ 0.05).

**TABLE 2b:** Heatmap of agreement across frailty cutoffs and indices (Cohen's kappa).

	mFI‐11 ≥ 0.36	mFI‐11 ≥ 0.27	mFI‐11 ≥ 0.18	mFI‐5 ≥ 0.40
mFI‐11 ≥0.36		0.642	0.297	0.427
mFI‐11 ≥0.27	0.642		0.539	0.572
mFI‐11 ≥0.18	0.297	0.539		0.510
mFI‐5 ≥0.40	0.427	0.572	0.510	

*Note*: This table is a heatmap of interdefinition agreement; cell shading visually represents the magnitude of Cohen's kappa (darker = stronger agreement) and is a visual aid only.

Median operative time ranged from 143 to 150 min across frailty definitions, and resected tissue weight ranged from 74 to 80 g, without significant between‐group differences. Postoperatively, haemoglobin on POD 1 was significantly lower in frail patients when defined by mFI‐11 ≥0.36 and ≥0.27, although haemoglobin change did not differ across definitions. Median length of stay was 1 day across all groups and was statistically longer only when frailty was defined using an mFI‐11 threshold of ≥0.27. Functional outcomes improved in both frail and nonfrail groups; however, frail patients defined by mFI‐11 ≥0.36 demonstrated significantly less improvement in IPSS at 1 year. Frail patients generally had lower Qmax during follow‐up. For example, at 3 months, median Qmax was 16.7 versus 20.1 ml/s in patients classified as frail versus nonfrail by mFI‐11 ≥0.36 (*p* = 0.01), and at 6 months, Qmax remained lower across all frailty definitions. In contrast, post‐void residual volumes did not show clinically meaningful differences between groups, although statistically significant differences were observed at 3 months for mFI‐11 ≥0.18, at 6 months for mFI‐5 ≥0.40, and for change in PVR at 12 months for mFI‐11 ≥0.27 (Table 3).

**TABLE 3:** Intraoperative, perioperative, and functional outcomes across different frailty cutoffs.

	mF11 ≥ 0.36			mF11 ≥ 0.27			mF11 ≥ 0.18			mF5 ≥ 0.4		
	Frail 139 (16.2%)	Nonfrail 718 (83.8%)	p	Frail 249 (29.1%)	Nonfrail 608 (70.9%)	p	Frail 451 (52.6%)	Nonfrail 406 (47.4%)	p	Frail 236 (27.5%)	Nonfrail 621 (72.5%)	p
**Intraoperative**												
Duration of surgery(min)	149 [110–187]	145 [106–197]	0.63	150 [111–194]	143 [104–197]	0.15	146 [105–192]	146 [107–200]	0.97	149 [108–190]	146 [106–199]	0.92
Resected amount (g)	74 [44–111]	79 [46–115]	0.57	74 [44–110]	80 [47–115]	0.34	76 [46–111]	80 [46–116]	0.78	78 [46–117]	78 [46–114]	0.45
**Perioperative and postoperative**												
Creatinine on POD1(mg/dL)	1.02 [0.88–1.24]	1.04 [0.90–1.18]	0.84	1.02 [0.88–1.21]	1.04 [0.90–1.19]	0.39	1.04 [0.89–1.21]	1.03 [0.91–1.18]	0.88	1.03 [0.88–1.21]	1.04 [0.90–1.19]	0.83
Hgb on POD 1 (g/dL)	11.8 [10.6–13.0]	12.2 [11.0–13.2]	**0.03**	11.9 [10.6–13.1]	12.2 [11.1–13.2]	**0.05**	12.1 [10.8–13.1]	12.2 [11.1–13.3]	0.09	11.9 [10.5–13.1]	12.2 [11.0–13.2]	0.06
Hgb change POD 1(g/dL)	2.0 [1.0–2.9]	1.9 [1.1–2.8]	0.92	2.0 [1.1–3.0]	1.8 [1.1–2.8]	0.58	1.9 [1.0–2.8]	1.9 [1.2–2.9]	0.57	2.0 [1.0–2.9]	1.9 [1.1–2.8]	0.93
Length of stay (days)	1 [1–1]	1 [1–1]	0.14	1 [1–1]	1 [1–1]	**0.02**	1 [1–1]	1 [1–1]	0.50	1 [1–1]	1 [1–1]	0.48
Catheterization (days)	1 [1–2]	1 [1–2]	0.55	1 [1–2]	1 [1–2]	0.73	1 [1–2]	1 [1–2]	0.98	1 [1–2]	1 [1–2]	0.92
Incidental Pca	19 (13.7%)	89 (12.4%)	0.67	31 (12.4%)	77 (12.7%)	1	61 (13.5%)	47 (11.6%)	0.41	31 (13.1%)	77 (12.4%)	0.82
PSA at 3 months	0.4 [0.2–0.7]	0.4 [0.2–0.7]	0.31	0.4 [0.2–0.7]	0.4 [0.2–0.7]	0.22	0.4 [0.2–0.7]	0.4 [0.2–0.7]	0.13	0.3 [0.2–0.7]	0.4 [0.2–0.7]	0.08
**IPSS**												
3 months	3 [1–6]	3 [1–6]	0.93	3 [1–6]	3 [1–6]	0.84	3 [1–6]	3 [1–6]	0.90	3 [1–6]	3 [1–6]	0.95
6 months	2 [0–4]	2 [0–4]	0.86	2 [0–4]	2 [0–4]	0.84	2 [0–4]	2 [0–4]	0.45	2 [0–4]	2 [0–4]	0.72
12 months	4 [0–5]	1 [0–3]	**0.03**	2 [0–4]	1 [0–3]	0.56	1 [0–3]	1 [0–4]	0.89	2 [0–4]	1 [0–3]	0.26
**Change in IPSS**												
3 months	14 [8–21]	13 [8–19]	0.24	13 [8–20]	13 [8–19]	0.57	13 [8–19]	13 [8–19]	0.89	12 [8–21]	13 [8–19]	0.75
6 months	16 [11–20]	15 [10–20]	0.94	16 [11–20]	15 [10–20]	0.73	16 [10–20]	15 [11–20]	0.36	16 [11–20]	15 [10–20]	0.96
12 months	18 [9–21]	15 [10–21]	0.77	18 [10–20]	14 [10–21]	0.70	15 [9–21]	15 [11–21]	0.99	17 [8–20]	15 [11–21]	0.72
**Qmax (mL/s)**												
3 months	16.7 [10.4–24.9]	20.1 [11.9–29.0]	**0.01**	17.0 [10.8–26.1]	20.1 [11.9–29.0]	0.06	18.9 [11.1‐27.3]	20.0 [11.5–29.1]	0.32	17.0 [11.0–25.0]	20.1 [11.9–29.1]	0.06
6 months	15.7 [9.4–22.6]	20.5 [12.2–29.3]	**0.01**	16.4 [9.7–24.0]	20.9 [12.4–29.9]	**0.01**	18.1 [11.0–28.1]	21.0 [12.4–30.4]	**0.05**	16.7 [10.0–25.0]	20.5 [12.2–29.9]	**0.01**
12 months	16.0 [10.0–26.9]	20.4 [13.3–30.0]	0.17	16.7 [11.0–28.5]	20.5 [13.4–30.1]	0.19	19.8 [12.9–29.2]	20.2 [12.7–30.0]	0.96	19.4 [10.9–26.9]	20.2 [13.4–30.1]	0.39
**Change in Qmax (mL/s)**												
3 months	7.5 [0.0–14.9]	11.7 [3.3–21.8]	**0.01**	8.8 [0.2–16.7]	12.0 [3.7–22.7]	**0.02**	10.1 [2.6–20.2]	11.7 [2.7–22.7]	0.40	8.3 [1.0–20.1]	11.7 [3.4–21.2]	0.12
6 months	7.0 [2.6–16.1]	12.5 [4.2–21.2]	0.10	7.1 [1.4–16.0]	13.0 [5.2–21.9]	**0.02**	10.1 [2.8–17.7]	12.9 [5.4–22.5]	0.10	10.6 [1.0–17.1]	11.4 [4.5–21.4]	0.11
12 months	6.7 [2.4–14.8]	12.2 [4.8–20.4]	0.06	7.8 [2.5–17.9]	12.6 [4.8–21.0]	**0.03**	9.8 [3.1–19.8]	11.8 [4.3–21.0]	0.39	8.5 [2.2–17.9]	11.9 [4.5–21.0]	0.12
**PVR (mL)**												
3 months	11 [0–48]	16 [2–45]	0.24	14 [0–52]	17 [2–45]	0.35	12 [0–44]	19 [3–46]	**0.01**	15 [0–46]	17 [1–45]	0.70
6 months	15 [0–54]	20 [1–60]	0.28	22 [0–70]	18 [1–53]	0.31	25 [1–62]	15 [1–53]	0.21	35 [2–73]	16 [1–51]	**0.03**
12 months	20 [1–47]	15 [0–42]	0.35	16 [2–48]	15 [0–42]	0.33	15 [0–40]	15 [0–43]	0.99	17 [0–36]	15 [0–43]	0.94
**Change in PVR (mL)**												
3 months	145 [15–264]	104 [26–238]	0.56	125 [23–279]	101 [25–228]	0.29	110 [22–251]	101 [31–230]	0.90	92 [17–248]	106 [30–245]	0.54
6 months	132 [32–290]	93 [10–212]	0.15	114 [14–246]	93 [11–212]	0.53	107 [8–240]	92 [17–206]	0.94	113 [5–241]	94 [14–221]	0.99
12 months	134 [40–458]	105 [20–227]	0.22	136 [34–449]	103 [20–212]	**0.05**	108 [19–276]	107 [27–220]	0.47	115 [36–291]	104 [20–222]	0.23

*Note*: Bold values are the *p* values that were statistically significant (defined as *p* ≤ 0.05).

Transfusion differed significantly only at the mFI‐11 ≥0.36 cutoff, occurring in 4.3% of frail patients versus 1.1% of nonfrail patients (*p* = 0.01). UTI requiring antibiotics demonstrated a significant dose–response relationship across increasing frailty strata (Tables 4a and 4b). In continuous modelling, higher mFI‐11 score was significantly linked with greater odds of UTI, corresponding to an approximately 20% increase in odds per 1‐point increase in mFI‐11, which is equivalent to an approximate 0.09 increase in the mFI‐11 index. In diagnostic performance analyses, only mFI‐11 ≥0.36 demonstrated discriminatory ability beyond chance for transfusion (Table 4b). By comparison, complication rates were broadly similar when stratified by mFI‐5 ≥0.40, and mFI‐5 did not demonstrate a consistent relationship with postoperative complications.

**TABLE 4a:** Complication rates across cutoffs and indices.

mFI‐11														
Complication	Cutoff = 0.18				Cutoff = 0.27				Cutoff = 0.36				Cochran Armitage	Logistic regression
	Frail 451 (52.6%)	Nonfrail 406 (47.4%)	*p*	OR (CI)	Frail 249 (29.1%)	Nonfrail 608 (70.9%)	*p*	OR (CI)	Frail 139 (16.2%)	Nonfrail 718 (83.8%)	*p*	OR (CI)		
**Major (CD ≥3)**	18 (4.0%)	17 (4.2%)	0.98	0.95 (0.48–1.87)	12 (4.8%)	23 (3.8%)	0.61	1.29 (0.63–2.63)	7 (5.0%)	28 (3.9%)	0.55	1.31 (0.56–3.05)	0.37	1.09 (0.90–1.32) 0.360
BNC	8 (1.8%)	9 (2.2%)	0.83	0.80 (0.30–2.08)	6 (2.4%)	11 (1.8%)	0.76	1.34 (0.49–3.66)	3 (2.2%)	14 (1.9%)	0.87	1.11 (0.32–3.91)	0.36	1.05 (0.80–1.38) 0.727
USD	9 (2.0%)	11 (2.7%)	0.64	0.73 (0.30–1.78)	6 (2.4%)	14 (2.3%)	0.99	1.05 (0.40–2.76)	3 (2.2%)	17 (2.4%)	0.88	0.91 (0.26–3.15)	0.48	0.99 (0.76–1.30) 0.958
**Minor (CD ≤2)**	256 (56.8%)	225 (55.4%)	0.74	1.06 (0.81–1.38)	142 (57.0%)	339 (55.8%)	0.79	1.05 (0.78–1.42)	78 (56.1%)	403 (56.1%)	0.99	1.00 (0.69–1.44)	0.17	1.00 (0.93–1.09) 0.943
Transfusion	7 (1.6%)	7 (1.7%)	0.99	0.90 (0.31–2.58)	7 (2.8%)	7 (1.2%)	0.15	2.48 (0.86–7.16)	6 (4.3%)	8 (1.1%)	**0.01**	4.00 (1.37–11.73)	0.07	1.22 (0.93–1.60) 0.142
AUR POD 1	37 (8.2%)	36 (8.8%)	0.82	0.92 (0.57–1.48)	23 (9.2%)	50 (8.2%)	0.73	1.14 (0.68–1.91)	12 (8.6%)	61 (8.5%)	0.96	1.02 (0.53–1.94)	0.31	1.04 (0.90–1.19) 0.612
AKI	5 (1.1%)	9 (2.2%)	0.31	0.49 (0.16–1.49)	3 (1.2%)	11 (1.8%)	0.74	0.66 (0.18–2.39)	2 (1.4%)	12 (1.7%)	0.84	0.86 (0.19–3.88)	0.32	0.92 (0.66–1.29) 0.641
UTI	35 (7.8%)	24 (5.9%)	0.35	1.34 (0.78–2.29)	23 (9.2%)	36 (5.9%)	0.11	1.62 (0.94–2.79)	14 (10.1%)	45 (6.3%)	0.10	1.68 (0.89–3.14)	**0.01**	**1.20 (1.04–1.38) 0.011**
TUI (<6 weeks)	234 (51.9%)	206 (50.7%)	0.79	1.05 (0.80–1.37)	131 (52.6%)	309 (50.8%)	0.69	1.07 (0.80–1.44)	79 (56.8%)	361 (50.3%)	0.16	1.30 (0.90–1.88)	0.19	1.04 (0.96–1.12) 0.381

mFI‐5					
Complications	Frail 236 (27.5%)	Nonfrail 621 (72.5%)	*p*	OR (CI)	Logistic regression
**Major (CD ≥3)**	10 (4.2%)	25 (4.0%)	0.85	1.05 (0.50–2.23)	1.12 (0.77–1.61); 0.557
BNC	6 (2.5%)	11 (1.8%)	0.58	1.45 (0.53–3.96)	1.24 (0.75–2.07); 0.404
USD	7 (3.0%)	10 (1.6%)	0.27	1.87 (0.70–4.97)	1.16 (0.69–1.95); 0.575
**Minor (CD ≤2)**	126 (53.4%)	354 (57.0%)	0.36	0.86 (0.64–1.17)	0.96 (0.83–1.12); 0.596
Transfusion	4 (1.7%)	10 (1.6%)	1	1.05 (0.33–3.39)	0.93 (0.51–1.70); 0.813
AUR POD 1	22 (9.3%)	51 (8.2%)	0.59	1.15 (0.68–1.94)	1.09 (0.84–1.42); 0.501
AKI	4 (1.7%)	10 (1.6%)	1	1.05 (0.33–3.39)	1.02 (0.57–1.84); 0.948
UTI	17 (7.2%)	42 (6.8%)	0.88	1.07 (0.60–1.92)	1.21 (0.91–1.61); 0.190
TIU (<6 weeks)	122 (51.7%)	318 (51.2%)	0.88	1.03 (0.76–1.39)	1.03 (0.89–1.20); 0.693

*Note*: Bold marks the statistically significant results (*p* ≤ 0.05): the significant within‐cutoff *p* value, the significant Cochran–Armitage trend *p* value, and the corresponding significant logistic‐regression odds ratio (95% CI excluding 1).

Abbreviations: AKI, acute kidney injury; AUR, acute urinary retention; BNC, bladder neck contracture; CD, Clavien–Dindo; TUI, transient urinary incontinence; USD, urethral stricture disease; UTI, urinary tract infection.

**TABLE 4b:** Diagnostic performance of mFI–5 and alternative mFI–11 cutoffs for complications after HoLEP.

Complication	mFI−11 cutoff = 0.18			mFI−11 cutoff = 0.27			mFI−11 cutoff = 0.36			mFI−5 cutoff = 0.40		
	Sensitivity (%)	Specificity (%)	*J*	Sensitivity (%)	Specificity (%)	*J*	Sensitivity (%)	Specificity (%)	*J*	Sensitivity (%)	Specificity (%)	*J*
**Major (CD ≥3)**	51.4 (34.0–68.6)	47.3 (43.9–50.8)	−0.01 (−0.19–0.16)	34.3 (19.1–52.2)	71.2 (67.9–74.2)	0.05 (−0.10–0.24)	20.0 (8.4–36.9)	83.9 (81.3–86.4)	0.04 (−0.08–0.21)	28.6 (14.6–46.3)	72.5 (69.3–75.5)	0.01 (−0.13–0.19)
BNC	47.1 (23.0–72.2)	47.3 (43.8–50.7)	−0.06 (−0.30–0.20)	35.3 (14.2–61.7)	71.1 (67.9–74.1)	0.06 (−0.15–0.33)	17.6 (3.8–43.4)	83.8 (81.1–86.2)	0.01 (−0.13–0.27)	35.3 (14.2–61.7)	72.6 (69.5–75.6)	0.08 (−0.13–0.34)
USD	45.0 (23.1–68.5)	47.2 (43.8–50.6)	−0.08 (−0.30–0.16)	30.0 (11.9–54.3)	71.0 (67.8–74.0)	0.01 (−0.17–0.25)	15.0 (3.2–37.9)	83.8 (81.1–86.2)	−0.01 (−0.13–0.22)	41.2 (18.4–67.1)	72.7 (69.6–75.7)	0.14 (−0.09–0.40)
**Minor (CD ≤2)**	53.2 (48.7–57.8)	48.1 (43.0–53.3)	0.01 (−0.06–0.08)	29.5 (25.5–33.8)	71.5 (66.7–76.1)	0.01 (−0.05–0.07)	16.2 (13.0–19.8)	83.8 (79.7–87.4)	0.00 (−0.05–0.05)	26.2 (22.4–30.4)	70.8 (65.9–75.4)	−0.03 (−0.09–0.03)
Transfusion	50.0 (23.0–77.0)	47.3 (43.9–50.8)	−0.03 (−0.30–0.25)	50.0 (23.0–77.0)	71.3 (68.1–74.3)	0.21 (−0.06–0.48)	42.9 (17.7–71.1)	84.2 (81.6–86.6)	**0.27** **(0.02–0.55)**	28.6 (8.4–58.1)	72.5 (69.3–75.5)	0.01 (−0.19–0.31)
AUR POD1	50.7 (38.7–62.6)	47.2 (43.7–50.8)	−0.02 (−0.15–0.10)	31.5 (21.1–43.4)	71.2 (67.9–74.3)	0.03 (−0.08–0.15)	16.4 (8.8–27.0)	83.8 (81.0–86.3)	0.00 (−0.08–0.11)	30.1 (19.9–42.0)	72.7 (69.4–75.8)	0.03 (−0.08–0.15)
AKI	35.7 (12.8–64.9)	47.1 (43.7–50.5)	−0.17 (−0.40–0.12)	21.4 (4.7–50.8)	70.8 (67.6–73.9)	−0.08 (−0.25–0.22)	14.3 (1.8–42.8)	83.7 (81.1–86.2)	−0.02 (−0.15–0.27)	28.6 (8.4–58.1)	72.5 (69.3–75.5)	0.01 (−0.19–0.31)
UTI	59.3 (45.7–71.9)	47.9 (44.4–51.4)	0.07 (−0.07–0.20)	39.0 (26.5–52.6)	71.7 (68.4–74.8)	0.11 (−0.02–0.25)	23.7 (13.6–36.6)	84.3 (81.6–86.8)	0.08 (−0.02–0.21)	28.8 (17.8–42.1)	72.6 (69.3–75.6)	0.01 (−0.10–0.15)

*Note*: Bold denotes the only Youden's *J* statistic whose 95% confidence interval excludes 0, indicating discrimination beyond chance.

Abbreviations: AKI, acute kidney injury; AUR, acute urinary retention; BNC, bladder neck contracture; CD, Clavien–Dindo; USD, urethral stricture disease; UTI, urinary tract infection.

## 4 DISCUSSION

Frailty provides a clinically relevant framework for risk assessment and has been linked to worse outcomes and slower recovery, including longer hospitalization, higher morbidity, and increased mortality across various populations.^12^, ^13^ Despite its relevance, frailty assessment is inconsistent across studies and multiple tools exist.^9^, ^26^ Although multiple frailty measures predict outcomes, concordance between measures is often modest.^26^ Among commonly used instruments, mFI is widely applied in surgical populations.^14^ Despite its widespread use, inconsistencies extend to threshold selection, as mFI‐11 cutoffs used in the literature commonly range from ≥0.18 to ≥0.36 and appear context‐dependent rather than universal.^14^, ^15^ In the context of BPO, literature remains limited and has predominantly relied on mFI‐5.^26^, ^27^ Our results indicate that HoLEP remains safe and effective across both indices and cutoffs, reinforcing the guidelines.^19^

Across our cohort, frailty burden was generally low with roughly two out of every three patients identified as nonfrail. In this cohort, common components such as hypertension and diabetes contributed substantially to the overall deficit burden, which may have influenced score distribution and reduced how distinctly frailty separated some groups. The proportion labelled ‘frail’ shifted substantially depending on the cutoff, which is consistent with literature showing that changing the frailty instrument or threshold meaningfully changes classification.^27^ This also mirrors broader cross‐specialty comparisons where mFI‐5 and mFI‐11 tend to produce different frailty prevalence despite tracking similar risk gradients.^28^ Agreement rates between various frailty assessment approaches were modest, further emphasizing the need for standardization. This observation aligns with Subramaniam et al.'s findings indicating low agreement rates between mFI‐11 and other frailty tools.^26^ Agreement was strongest between the more restrictive mFI‐11 thresholds, reaching the range typically considered substantial (*κ* 0.60–0.80), while no pairing achieved near‐perfect agreement (*κ* > 0.80), highlighting that ‘frail’ is not interchangeable across cutoffs and indices.

Baseline characteristics were largely comparable. Frail patients were significantly older, consistent with Ren et al.'s findings.^21^ Because frail patients were older across all definitions, some observed differences may partly reflect age‐related physiologic changes in bladder dysfunction in addition to frailty. Nonfrail patients had higher baseline PSA than frail patients, contrasting Ren et al. who did not find a significance difference.^18^ Preoperative haemoglobin differed significantly only when frailty was defined using the strictest threshold (mFI‐11 ≥0.36), suggesting that anaemia may cluster among patients with the highest burden. In our HoLEP cohort, preoperative catheter dependency was significantly more common in frail patients when frailty was defined more broadly, but this pattern diminished as the cutoff got stricter.

Intraoperative and perioperative outcomes were largely comparable between groups. Haemoglobin on POD 1 was significantly lower with stricter frailty definitions and approached significance with broader cutoffs, whereas the change in haemoglobin did not differ meaningfully across definitions. This pattern may reflect baseline differences in preoperative haemoglobin, with lower starting values more common among the frailest. Length of stay differed only when frailty was defined as mFI‐11 ≥0.27. This may reflect differences in group size and case‐mix across thresholds and should be interpreted cautiously.

Our functional findings suggest that HoLEP preserves meaningful functional improvements even among frail individuals, with statistically significant differences appearing only at selected time points and depending on the frailty definition. This finding aligns with Balsamo et al., who showed frail patients demonstrating functional improvement with Rezum but contrasts with TURP‐based frailty literature, in which Ren et al. reported frailty as a risk marker for less favourable outcomes and a blunted improvement.^18^, ^24^ This divergence implies that traditional resection may magnify frailty‐related disparities in recovery. In our cohort, symptom improvement was observed across frailty groups, and changes in IPSS did not differ significantly across definitions. At the same time, our observation that Qmax improvement was generally less pronounced among frail patients across definitions aligns with Ren et al.^18^ Though frailty and age do not overlap perfectly, frail patients are often older. Although frail patients had lower Qmax at selected time points, this difference may partly reflect older age and baseline bladder dysfunction rather than frailty alone.^29^

Overall complication rates were low for major events, and neither index nor cutoff showed a consistent gradient for major complications. Minor complications were numerically common, largely driven by TIU, which likely reflects our broad definition of any leakage including stress or urge episodes requiring even minimal pad use. This broad definition may have diluted clinically meaningful differences between frailty groups. Within‐cutoff comparisons identified a difference in transfusion rates only at the strictest mFI‐11 ≥0.36 threshold, which is clinically coherent given that this group also had lower baseline and early postoperative haemoglobin. UTI requiring antibiotics was the only complication to demonstrate a consistent association with increasing mFI‐11. In continuous modelling, each 1‐point increase in mFI‐11 score was associated with an approximately 20% increase in the odds of UTI. However, this finding should be interpreted cautiously, as effect sizes across dichotomized cutoffs were modest. Though age does not translate into frailty, it is still not surprising that the strongest signal emerged for UTI, as UTI‐related hospitalizations among the elderly have continued to rise even as admissions for many other conditions decline.^30^ This likely reflects the convergence of bladder outlet obstruction and catheter exposure with the heightened susceptibility to infection by frailty‐associated immunosenescence. By comparison, mFI‐5 did not yield a consistent complication signal, suggesting that the added granularity of mFI‐11 may better capture vulnerability. This relationship with complications differs from broader surgical literature in which frailty has been linked to postoperative morbidity, longer hospitalization, and higher overall complication burden.^12^, ^13^ This difference may reflect HoLEP's safety profile in medically complex patients.

Across the evaluated thresholds and indices, diagnostic discrimination for postoperative complications was generally limited. Lower mFI‐11 cutoffs tended to yield higher sensitivity but at the expense of specificity, whereas the stricter cutoffs improved specificity with lower sensitivity. mFI‐5 ≥0.40 demonstrated high sensitivity but poor specificity across outcomes. The only clinically notable signal was observed for transfusion, where mFI‐11 ≥0.36 showed meaningful discriminatory ability.

In conclusion, HoLEP remained safe and effective across frailty definitions, with functional improvement observed irrespective of the index or cutoff. However, the choice of frailty threshold meaningfully influenced risk discrimination. Within the HoLEP context, mFI‐11 ≥0.36 appeared to provide the most clinically informative stratification, with mFI‐11 ≥0.27 performing similarly and potentially serving as a pragmatic alternative. When full mFI‐11 calculation is impractical, mFI‐5 remains a reasonable screening tool for HoLEP candidacy, although it may be less sensitive. In contrast, mFI‐11 ≥0.18 classified a large proportion of patients as frail and may be overly conservative for HoLEP, potentially underestimating the widely acknowledged safety profile of HoLEP.

This study has several limitations. First, its retrospective design introduces inherent risks of bias. Second, HoLEP is a highly standardized, minimally invasive procedure with a favourable safety profile, which may attenuate frailty‐related differences and limit generalizability of our findings to other BPO procedures. Third, frailty was assessed exclusively using mFI‐based definitions; inclusion of alternative frailty instruments capturing complementary domains could have strengthened external validity and comparability. Finally, this was a single‐institution, single‐surgeon study, further limiting its external applicability.

## 5 CONCLUSION

Across frailty indices and cutoffs, HoLEP remained safe and effective, with meaningful functional improvement observed in both groups. Major complications were uncommon and did not show a consistent frailty gradient. Transfusion differed only at the strictest mFI‐11 threshold (≥0.36), and UTI demonstrated a dose–response relationship and approximately 20% increase in odds per 1‐score increase (0.09 index) with mFI‐11. Within the HoLEP context, mFI‐11 ≥0.36 appeared to offer the most informative stratification, while mFI‐11 ≥0.27 may serve as a pragmatic alternative. In contrast, mFI‐11 ≥0.18 classified a large proportion as frail and may be overly conservative for HoLEP.

## AUTHOR CONTRIBUTIONS

**Hasim Bakbak:** Conceptualization; data curation; investigation; methodology; visualization; writing—original draft; writing—review and editing. **Gurpremjit Singh:** Methodology; validation; visualization; writing—review and editing. **Ahmad Abdelaziz:** Conceptualization; investigation; validation; writing—review and editing. **Adam Williams:** Formal analysis; writing—review and editing. **Juanita Velasquez:** Data curation; investigation; writing—review and editing. **Jonathan E. Katz:** Validation; writing—review and editing. **Robert Marcovich:** Validation; writing—review and editing. **Alan Jerome Wein:** Validation; writing—review and editing. **Hemendra N. Shah:** Conceptualization; project administration; resources; supervision; writing—review and editing.

## CONFLICT OF INTEREST STATEMENT

The authors declare that the research was conducted in the absence of any commercial or financial relationships that could be construed as a potential conflict of interest.

## Data Availability

The raw data supporting the conclusions of this article will be made available by the authors, without undue reservation, upon reasonable request and after approval by the institutional review board.
